# Rural–Urban Variability in Home and Community‐Based Service Use Among Veterans

**DOI:** 10.1111/jgs.70500

**Published:** 2026-06-10

**Authors:** Heather Davila, Daniel Hackert, Frank DeVone, Christopher W. Halladay, Michelle A. Mengeling, Samantha L. Solimeo, R. Neal Axon, Mia L. Barron, Casey Buchanan, Tonya Page, Dayna Cooper, Jennifer L. Sullivan, James L. Rudolph

**Affiliations:** ^1^ Center for Access & Delivery Research and Evaluation (CADRE) Iowa City VA Health Care System Iowa City Iowa USA; ^2^ VHA Office of Rural Health, Veterans Rural Health Resource Center‐Iowa City Iowa City VA Health Care System Iowa City Iowa USA; ^3^ Department of Internal Medicine University of Iowa Carver College of Medicine Iowa City Iowa USA; ^4^ Transformative Health Systems Research to Improve Veteran Equity and Independence (THRIVE) VA Providence Healthcare System Providence Rhode Island USA; ^5^ Health Equity and Rural Outreach Innovation Center (HEROIC) Ralph H. Johnson VA Health Care System Charleston South Carolina USA; ^6^ Department of Medicine Medical University of South Carolina Charleston South Carolina USA; ^7^ Veterans Affairs Quality Scholars Ralph H. Johnson VA Medical Center Charleston South Carolina USA; ^8^ Office of Geriatrics and Extended Care, U.S. Department of Veterans Affairs Washington, DC USA; ^9^ Department of Health Services, Policy and Practice, School of Public Health Brown University Providence Rhode Island USA; ^10^ Department of Medicine, Warren Alpert School of Medicine Brown University Providence Rhode Island USA

**Keywords:** access to care, aging, home and community‐based services, rural healthcare

## Abstract

**Background:**

Home and community‐based services (HCBS) are home‐based services that enable people to remain in their own environment despite challenges related to disease, disability, or age. In rural areas, service availability may be lower. The purpose of this analysis was to examine rural–urban differences in HCBS use among Veterans enrolled in the Veterans Health Administration (VHA) and identify facility‐level variation.

**Methods:**

This cross‐sectional study used data from fiscal year 2022 (10/1/21–9/30/22). We incorporated rurality for Veterans (dichotomized as rural/urban based on Rural–Urban Area Commuting Codes) and facilities (proportion of rural Veterans served). VHA payment files identified HCBS use. Regression analyses sequentially adjusted for demographics, comorbidity, Area Deprivation Index (ADI), and facility fixed effects to produce a risk ratio (RR) of HCBS use among rural Veterans (RR > 1.0 indicates higher HCBS use among rural vs. urban Veterans).

**Results:**

Of over 6 million enrolled Veterans, 34.1% (*n* = 2,055,746) were identified as rural. Compared to urban Veterans, rural Veterans were more likely to be older (64.1 ± 16.1 vs. 61.0 ± 17.4 years, *p* < 0.001), male (92.0% vs. 88.8%, *p* < 0.001), and white (80.5% vs. 63.7%, *p* < 0.001). HCBS were used by 5.04% (*n* = 103,605) of rural Veterans and 5.21% (*n* = 206,608) of urban Veterans. The unadjusted rural HCBS RR was 0.97 (95% confidence interval (CI) = 0.96–0.97). After adjusting for demographics, comorbidity, ADI, and facility fixed effects, the adjusted RR of HCBS for rural Veterans was 0.92 (95% CI = 0.91–0.93). There was substantial variability across facilities, with rural Veterans ranging from 60% less likely to 167% more likely to use HCBS than urban Veterans (RRs: 0.40–2.67).

**Conclusion:**

While rural Veterans were less likely to use HCBS overall, there was substantial variability across facilities. These findings demonstrate that some VAMCs counteract the overall trend by ensuring rural Veterans receive HCBS at rates comparable to urban Veterans.

## Summary

- Key Points
  ○ Rural Veterans were 8% less likely than urban Veterans to use home‐ and community‐based services (HCBS) after adjusting for demographics, comorbidity, area economic disadvantage, and facility fixed effects.
  ○ Although HCBS use was lower among rural Veterans overall, there was wide variation across facilities.
  ○ Learning from facilities where rural and urban Veterans use HCBS at similar rates could help inform strategies to improve HCBS delivery nationally.
- Why does this paper matter?
  ○ This study highlights meaningful rural–urban differences in HCBS use among Veterans, along with substantial variation across medical centers. Identifying facilities where rural and urban Veterans use HCBS equitably may inform actionable strategies to improve access nationwide.

## 1 Introduction

Expanding Veterans' access to home‐ and community‐based services (HCBS) is a major priority of the U.S. Department of Veterans Affairs (VA), Veterans Health Administration (VHA) [1, 2]. HCBS are critical to preserving aging in place, a key goal of most older people, including Veterans. These services are especially important for rural Veterans, who are older than urban Veterans and who have higher rates of chronic illness, social isolation, and mobility limitations [3, 4]. VHA offers a range of HCBS to meet the needs of aging Veterans and those with disabilities. The majority of these services are provided by contract community agencies, which also deliver HCBS to individuals covered by Medicare, Medicaid, or private insurance. Despite differing payer models, with the higher prevalence of advanced age, comorbidity, and disability among the Veteran population [5], delivery of HCBS within the VHA may serve as a harbinger of HCBS delivery nationally, particularly in hard‐to‐access geographic areas.

Outside the VHA, rural adults report lower use of personal care services [6, 7], home health [6, 8], and adult day services [6, 9] relative to their urban counterparts, with differences in personal care and adult day services particularly marked. Conversely, rural residents have reported a greater likelihood of using targeted case management [6], transportation services [6], and home‐delivered meals [7, 9], and similar to lower unmet HCBS needs than their urban counterparts [10]. Parikh et al. found that during the COVID‐19 public health emergency (2020–2021), adults overall reported increased use of personal care and caregiver support services, decreased use of homemaker/chore and adult day services, and higher unmet HCBS needs [7]. While our study focuses on HCBS use in the post‐COVID period, these earlier findings provide important context by highlighting disruptions in service availability and use that may have shaped subsequent patterns of HCBS access and need.

Rural areas present unique challenges to HCBS delivery. Within the VHA, staff and leaders have described workforce, transportation, and infrastructure barriers to meeting rural Veterans' HCBS needs [11, 12]. One significant issue is the extended drive times and transportation difficulties in many rural areas, which can hinder access to these essential services [11, 12, 13]. Additionally, the limited scale in rural areas, where only a few individuals may require services, can pose logistical problems for service delivery [13, 14, 15], as well as barriers to establishing and maintaining service contracts between VHA and community agencies, particularly for smaller agencies with fewer resources to manage such relationships [16]. The economic challenges of the above can result in a shortage of agencies and a reduced workforce [11, 14, 17], compounded by financial barriers, making it difficult to provide comprehensive HCBS. Such barriers are partially mitigated through direct contracting, which enables VAMCs to purchase HCBS from local community providers when existing network coverage is insufficient [18], as well as through VHA's Veteran‐directed care program, which enables Veterans to hire their own caregivers [19, 20]. However, community providers are not always available, and the relatively recent and uneven adoption of Veteran‐directed care limits its overall reach [19, 20].

Outside VHA, insurance coverage for HCBS is often limited. While Medicare covers home care, it is typically time‐restricted [21]. Medicaid also provides coverage, but there are often onerous requirements and limitations, plus waitlists in many states [22, 23]. Many older individuals are also reluctant or unable to pay out‐of‐pocket for HCBS, adding another layer of complexity [24, 25]. These multifaceted barriers collectively underscore the need for tailored strategies to improve HCBS delivery in rural populations.

The VHA is uniquely positioned as both a payer and provider of HCBS, enabling it to implement and evaluate innovative care models at scale. Unlike most healthcare systems, VHA integrates medical and long‐term care services under a unified structure, allowing coordinated delivery tailored to the complex needs of older Veterans. Improving rural Veterans' access to HCBS is a major policy priority, emphasized in the 2021 National Planning Strategy for Geriatrics [26]. Further, recent federal legislation—the Maintaining Internal Systems and Strengthening Integrated Outside Networks (MISSION) Act of 2018 [27] and the Elizabeth Dole 21st Century Veterans Healthcare and Benefits Improvement Act of 2025 [28]—has accelerated VHA investments in community‐based care, particularly for rural Veterans and others who experience barriers to accessing needed services. Its national reach, centralized data infrastructure, and mission‐driven focus on ensuring access to care for rural and other hard‐to‐reach Veterans make the VHA synergistic for advancing HCBS delivery. At the same time, HCBS delivery in VHA is also influenced by local leadership priorities, internal staffing capacity, and the availability of community‐based partners to deliver HCBS [29]. Thus, differences in HCBS availability and use are expected across the VHA system. As the US grapples with how to meet the growing demand for HCBS, the VHA's experience can inform broader efforts to expand HCBS access nationally.

To better understand variation in HCBS use among rural Veterans, we conducted an analysis of 2022 national VHA data to examine rurality‐related differences in HCBS use among Veterans and to identify facility‐level variation. Identifying these patterns can support learning from high‐performing sites and inform targeted quality improvement efforts. We hypothesized that, among VHA enrollees, rural Veterans would be less likely than urban Veterans to receive HCBS but that there would be substantial variability across VA medical centers (VAMCs).

## 2 Methods

### 2.1 Cohort Identification

We used VHA's data infrastructure to identify the cohort of Veterans enrolled in VHA healthcare during fiscal year 2022 (10/1/21–9/30/22). Veterans who died prior to the start of fiscal year 2022 were excluded from the analysis (*N* = 620,544). Veterans were identified by their primary residential address and assigned rurality using Rural–Urban Commuting Area (RUCA) codes (2010 version), a classification system developed by the U.S. Departments of Agriculture and Health and Human Services that uses census tract data to define rurality based on population density, level of urbanization, and daily commuting patterns [30]. We used the definition of rurality established by the VA Planning Systems Support Group database and Office of Rural Health. Specifically, VA classifies census tracts with RUCA scores of 1.0 or 1.1 as urban, census tracts with RUCA scores of 10.0 as highly rural, and all other RUCA scores (2.0 through 9.0, and 10.1–10.3) as rural [31]. In this framework, urban areas are defined by a central urban core where most workers commute locally, while rural areas include small towns and micropolitan regions where residents typically work within their communities. Highly rural areas are the most remote, with fewer than 10% of workers commuting to an urban area [30]. We grouped rural and highly rural Veterans for our primary analysis, given the relatively small proportion of highly rural Veterans in the cohort (3.8%; *n* = 226,124). This work was classified as non‐research and conducted in support of internal operations in accordance with VHA Program Guide 1200.21.

We linked data from the VHA Corporate Data Warehouse, a national repository of patient and facility data, with claims data from the Integrated Veteran Care Consolidated Dataset for VHA‐paid care in the community, to identify our primary outcome: Veterans receiving HCBS during 2022. For this analysis, HCBS included home‐based primary care, homemaker/home health aide care, respite care, community adult day healthcare, skilled home health services, and Veteran‐directed care. A description of these services and how they are identified in VHA data is provided in Supporting Information. Home‐based primary care is delivered directly within the VHA system and Veteran‐directed care enables Veterans to hire their own caregivers. The VHA pays for the other HCBS, which are predominantly provided through contract agencies in the community. For each Veteran, the outcome was receipt of any HCBS.

### 2.2 Facility Rurality

We assigned each Veteran to a single VAMC based on geographic proximity. If a Veteran's HCBS was paid for by a different VAMC, the Veteran was assigned to that VAMC instead. This method allowed for the assignment of Veterans with migratory primary addresses (“snowbirds”) and those within the shoulder regions of distributions to be attributed to the VAMC responsible for the costs. For each VAMC, we calculated facility rurality as the percentage of Veterans who were rural or highly rural.

### 2.3 Covariates

From the VHA demographics file, we collected information on Veterans' age, sex, race/ethnicity, marital status, and rurality. We used the Elixhauser Comorbidity Index to quantify Veterans' comorbidity burden based on inpatient and outpatient encounter codes from 2021 [32, 33]. We included the tract‐level Area Deprivation Index (ADI) [34]. The ADI is a location‐derived variable that includes several features of area socioeconomic disadvantage, such as unemployment and poor housing quality. ADI scores range from 0 to 100, with higher scores indicating greater socioeconomic disadvantage and scores ≥ 85 often used to identify high‐risk vulnerable populations [35].

### 2.4 Analyses

Our first analysis compared demographic characteristics, ADI, and comorbidity for rural and urban Veterans. Because *p* values can be misleading in large samples—often indicating statistical significance for differences of minimal practical relevance—we calculated standardized mean differences (SMDs) for each comparison. An SMD of ≤ 0.20 is considered a small difference; SMDs of 0.50 and 0.80 are considered medium and large differences, respectively [36]. We then calculated the risk ratio (RR) for rural Veterans' HCBS use relative to urban Veterans, with a 95% confidence interval (CI) for the full cohort. Given the convergence challenges often encountered with log‐binomial models and because our analysis included all VAMCs to identify facility‐specific estimates, we used modified Poisson regression with robust standard errors to estimate adjusted RR [37]. Models adjusted for demographics, ADI, comorbidity, and included a facility fixed effect to account for time‐invariant facility characteristics (e.g., leadership priorities, facility culture, social work staffing, community provider availability). Veterans with missing data on model covariates were excluded from the modeling analysis (13.1%). Model fit was acceptable, as indicated by a McFadden pseudo *R* ^2^ of 0.18. In sensitivity analyses, rural and highly rural categories were modeled separately.

Next, for each VAMC, we assessed the association between facility rurality and the facility‐specific adjusted RR for HCBS use among rural vs. urban Veterans using Pearson's correlation coefficient. We excluded 4 facilities from this analysis because these VAMCs served fewer than 1% rural Veterans (3 VAMCs) or fewer than 1% urban Veterans (1 VAMC). For graphing purposes, we grouped VAMCs into five rurality categories (1%–10%, 11%–30%, 31%–50%, 51%–70%, and ≥ 71%) to create clear, interpretable groups that span the full range of facility rurality and allow meaningful visual comparison. The number of Veterans who received HCBS ranged from ≈325 to > 8000 Veterans per VAMC.

Finally, we examined the characteristics of VAMCs with rural HCBS use RRs ≥ 1.0 (i.e., VAMCs with equal or higher HCBS use among rural vs. urban Veterans) vs. VAMCs with RRs < 1.0. We compared the two groups by geographic region (West, Midwest, South, Northeast), VA complexity level, and the mean percentage of rural and highly rural Veterans enrolled by VAMC, using chi‐square tests for categorical variables and *t*‐tests for continuous variables. VAMCs are classified into five complexity levels (1a, 1b, 1c, 2, 3) based on patient acuity, service offerings, and clinical, research, and teaching missions [38]. Higher‐complexity facilities provide more specialized services and manage higher‐risk patient populations.

## 3 Results

### 3.1 Demographic, ADI, and Comorbidity Comparisons

The cohort included 6,020,094 Veterans, with 34% (2,055,746) residing in rural areas, while the remaining 66% (3,964,348) were urban residents (Table 1). Rural Veterans tended to be older (64.1 ± 16.1 years) than urban Veterans (61.0 ± 17.4 years, *p* < 0.001, SMD 0.18) and they were more likely to be male (92.0% vs. 88.8%, *p* < 0.001, SMD 0.11), white (80.5% vs. 63.7%, *p* < 0.001, SMD 0.44), and married (62.0% vs. 53.7%, *p* < 0.001, SMD 0.17). Rural Veterans lived in areas with considerably higher ADI scores than urban Veterans (rural mean = 63.73 vs. urban mean = 49.26, *p* < 0.001, SMD = 0.60).

**TABLE 1:** Veterans' demographic, area deprivation index, and clinical characteristics by rurality.

Characteristic	Rural *N* = 2,055,746	Urban *N* = 3,964,348	*p*	SMD
Age (Mean (SD))	64.1 (16.1)	61.0 (17.4)	< 0.001	0.180
Male sex	1,890,887 (92.0)	3,518,691 (88.8)	< 0.001	0.109
Race			< 0.001	0.439
White	1,654,596 (80.5)	2,526,555 (63.7)		
Black	177,127 (8.6)	890,086 (22.5)		
All Other	57,262 (2.8)	175,971 (4.4)		
Hispanic	70,743 (3.4)	362,747 (9.2)	< 0.001	0.239
Married	1,227,537 (62.0)	2,023,512 (53.7)	< 0.001	0.170
Area deprivation index (Mean (SD))	63.73 (22.23)	49.26 (25.84)	< 0.001	0.600
Elixhauser comorbidity index (Mean (SD))	2.03 (2.08)	1.94 (2.15)	< 0.001	0.042
Specific comorbidities				
Hypertension	1,694,232 (42.7)	1,026,558 (49.9)	< 0.001	0.145
Depression	767,278 (19.4)	363,666 (17.7)	< 0.001	0.043
Diabetes	759,482 (19.2)	456,169 (22.2)	< 0.001	0.075
Diabetes, complicated	493,489 (12.4)	293,948 (14.3)	< 0.001	0.054
Obesity	549,604 (13.9)	305,642 (14.9)	< 0.001	0.029
Pulmonary disease	462,926 (11.7)	313,575 (15.3)	< 0.001	0.105
Psychoses	281,803 (7.1)	120,597 (5.9)	< 0.001	0.05
Anemia	331,190 (8.4)	172,330 (8.4)	0.229	0.001
Alcohol use disorder	266,568 (6.7)	116,798 (5.7)	< 0.001	0.043
Hypothyroidism	257,433 (6.5)	170,652 (8.3)	< 0.001	0.069
Neurodegenerative disorder	271,724 (6.9)	153,514 (7.5)	< 0.001	0.024
Tumor	238,489 (6.0)	137,385 (6.7)	< 0.001	0.027
Fluid and electrolyte disorders	217,932 (5.5)	118,202 (5.7)	< 0.001	0.011
Peripheral vascular disease	211,519 (5.3)	142,454 (6.9)	< 0.001	0.066
Substance use disorder	183,147 (4.6)	69,869 (3.4)	< 0.001	0.062
Congestive heart failure	181,261 (4.6)	112,531 (5.5)	< 0.001	0.041
Stroke	75,787 (3.7)	137,885 (3.5)	< 0.001	0.011
Valvular disease	117,598 (3.0)	82,526 (4.0)	< 0.001	0.057
Dementia	55,609 (2.7)	116,197 (2.9)	< 0.001	0.014

*Note:* The Area Deprivation Index (ADI) ranks geographic areas from 1 to 100 based on various socioeconomic factors; higher numbers indicate greater socioeconomic disadvantage. ADI mean and median values for both rural and urban Veterans differed by less than 5%. Rural median: 67.0 (IQR: 48.0, 82.0); urban median: 48.0 (IQR: 29.0, 69.0). The Elixhauser Comorbidity Index (ECI) was used to capture patients' comorbidities using 30+ chronic conditions identified in their 2021 inpatient and outpatient clinical records [31, 32]. In addition to the overall ECI score, the table reports the most common ECI comorbidities present in the cohort (i.e., those affecting > 2% of the cohort).

Common comorbidities included diabetes, depression, hypertension, and obesity. Comorbidities were similar across groups, although rural Veterans were more likely to be diagnosed with substance use disorders, depression, and psychoses; urban Veterans were more likely to be diagnosed with hypertension, hypothyroidism, and pulmonary conditions (all SMD < 0.20).

### 3.2 Rural–Urban Differences in Veterans' Use of HCBS

Differences in HCBS use among rural versus urban Veterans are presented in Table 2. In unadjusted analyses, rural Veterans were slightly less likely to receive HCBS than urban Veterans: 5.04% of rural Veterans versus 5.21% of urban Veterans (RR = 0.97; 95% CI: 0.96–0.97). After adjusting for demographics, ADI, comorbidity, and facility fixed effects, the rural–urban disparity in HCBS use widened, with rural Veterans 8% less likely to use HCBS than urban Veterans (adjusted RR = 0.92; 95% CI: 0.91–0.93). The sensitivity analysis indicated considerably lower HCBS use among highly rural Veterans compared to their urban counterparts (adjusted RR: 0.84; 95% CI: 0.82–0.86).

**TABLE 2:** Relative risk for home‐ and community‐based service use among rural veterans.

	Rural *N* (%)	Urban veterans *N* (%)	RR for rural HCBS use (95% CI)	Adjusted RR for rural HCBS use^a^ (95% CI)
HCBS	103,605 (5.04)	206,608 (5.21)	0.97 (0.96–0.97)	0.92 (0.91–0.93)
No HCBS	1,952,141 (94.96)	3,757,740 (94.79)		

*Note:* In a sensitivity analysis that included two levels of rurality (rural and highly rural), rural Veterans had an adjusted RR of 0.93 (95% CI: 0.92–0.94), whereas highly rural Veterans had an adjusted RR of 0.84 (95% CI: 0.82–0.86).

Abbreviations: CI = confidence interval; HCBS = home and community‐based services; RR = relative risk.

^a^ Adjusted for demographics, Area Deprivation Index, Elixhauser comorbidities [31, 32], and facility fixed effects.

### 3.3 Variability in Rural Versus Urban HCBS Use by Facility Rurality

**FIGURE 1:**
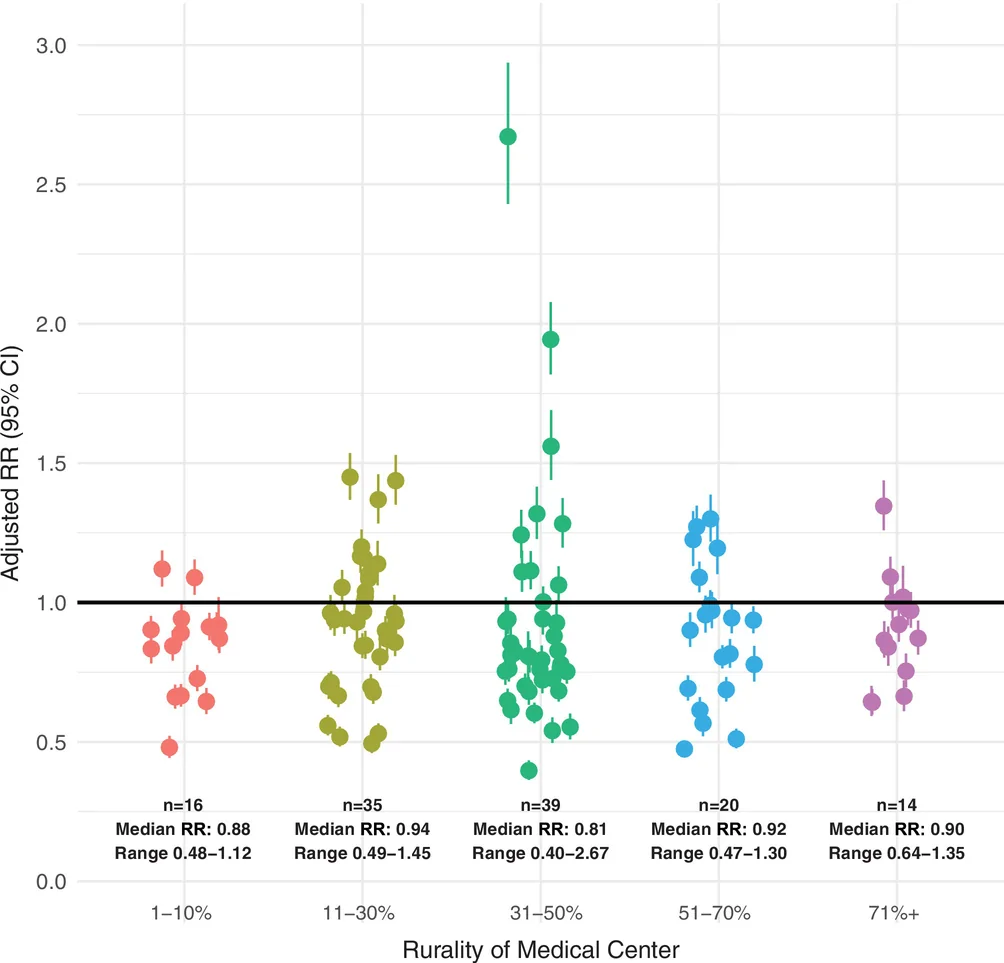
Adjusted facility risk ratios for home‐ and community‐based service use by rurality. Each dot represents one Veterans Affairs Medical Center (VAMC). VAMCs were grouped into five categories based on the percentage of rural Veterans served at the VAMC (facility rurality): 1%–10% rural, 11%–30%, 31%–50%, 51%–70%, and ≥ 71%. Four VAMCs were excluded from the facility‐level analysis because fewer than 1% of enrolled Veterans at the site were rural residents (3 VAMCs) or fewer than 1% of enrolled Veterans at the site were urban residents (1 VAMC). Lower adjusted risk ratio (aRR) = rural Veterans were less likely than urban Veterans to receive HCBS at that VAMC. RRs were adjusted for demographic and clinical characteristics, Area Deprivation Index, and facility fixed effects. The number of Veterans who received HCBS ranged from ≈325 to > 8000 Veterans per VAMC.

We found substantial variability in adjusted RRs across VAMCs by facility rurality (Figure 1) (range: low RR = 0.40, high RR = 2.67). There was a negligible correlation between facility rurality and the adjusted RR for rural HCBS use, with the estimate near zero (*r* = 0.06; 95% CI: −0.12 to 0.24). However, for each rural category, there were VAMCs that overcame the challenges of rural HCBS delivery, with equal or higher rates of HCBS use among rural versus urban Veterans. As shown in Table 3, geographic region was the only characteristic that demonstrated a statistically significant difference between VAMCs with adjusted rural HCBS use RRs ≥ 1.0 versus < 1.0. For example, over half (19 of 36 VAMCs, 52.8%) of VAMCs in the West had RRs ≥ 1.0 compared to only 9.1% (3 of 33 VAMCs) of VAMCs in the Northeast.

**TABLE 3:** Comparison of facility characteristics for VAMCs where the adjusted risk ratio for rural HCBS use was ≥ 1 versus < 1.

	Facilities where RR ≥ 1.0 (*n* = 34)	Facilities where RR < 1.0 (*n* = 90)	*p*
Characteristic			
Region			< 0.001
West	19	17	
Midwest	7	21	
South	5	21	
Northeast	3	30	
Facility complexity level			0.44
1a—High complexity	8	30	
1b—High complexity	7	17	
1c—High complexity	4	15	
2—Medium complexity	6	10	
3—Low complexity	8	18	
Percent rural Veterans, mean (SD)	40.70%	37.00%	0.39
Percent highly rural Veterans, mean (SD)	5.40%	4.00%	0.21
Rural VAMC (≥ 50% rural patients)	10 (29.4%)	26 (28.8%)	1.0

*Note:* Regions are based on US Census Bureau regions. VAMCs are classified into five complexity levels (1a, 1b, 1c, 2, 3) based on patient acuity, service offerings, and clinical, research, and teaching missions [37]. Higher‐complexity facilities provide more specialized services and manage higher‐risk patient populations. One VAMC is not included in the regions listed above and 1 VAMC was missing facility complexity.

## 4 Discussion

Because there are physical and structural barriers to HCBS delivery in rural areas, we conducted an analysis of rural vs. urban HCBS use in a nationwide integrated healthcare system. Rural Veterans were slightly less likely to receive HCBS than urban Veterans, but this difference widened after adjusting for population demographics, comorbidity, ADI, and facility fixed effects. Importantly, at each level of facility rurality, there were some VAMCs at which rural Veterans used HCBS at similar or higher rates than urban Veterans. Learning from these VAMCs is critical to enhancing HCBS delivery to all rural areas.

Consistent with prior studies, our findings highlight the challenges of delivering HCBS to rural Veterans, showing modest but significantly lower rates of HCBS utilization among rural Veterans [39, 40, 41]. Our analysis adds important facility‐level measurement to the equation by characterizing significant variation in HCBS use across VAMCs and identifying locations that appear to have overcome the challenges of delivering rural HCBS. Such facility‐level measurement could be used in a performance dashboard by the VHA's Offices of Rural Health and Geriatrics and Extended Care to monitor rural–urban differences in HCBS use. Given evidence outside VHA that the magnitude and direction of rural–urban differences in service use vary substantially by type of HCBS [6, 7, 10], such a dashboard could also include different categories of HCBS (e.g., personal care services vs. more medically oriented HCBS or HCBS delivered directly by VHA versus HCBS delivered through contract community agencies).

Our finding that the rural HCBS disparity widened after risk adjustment highlights that system‐ or structural‐level factors likely contribute to the expanded rural–urban gap in HCBS use. In our analysis, the higher prevalence of factors associated with greater HCBS need among rural Veterans (e.g., older age and higher comorbidity burden) initially masked the underlying association with rural residence. Once these factors were adjusted for, the independent rural–urban difference became more apparent. In practice, extra attention may be warranted to ensure rural Veterans' HCBS needs are being addressed.

Studies exploring barriers and facilitators to HCBS use consistently describe community‐ and system‐level obstacles to participation [13, 29, 42, 43], while also identifying individual‐level barriers [29, 44]. If delivered within the context of overarching care coordination programs similar to services provided within VHA, HCBS are associated with significantly higher satisfaction among HCBS recipients and caregivers [45], as well as other improved outcomes, including longer institutionalization‐free survival [44]. Evidence suggests that effective care coordination plays a critical role in improving access to HCBS and reducing unmet HCBS needs, in part by helping individuals navigate fragmented service systems and connect with available in‐home supports [46, 47]. Effective HCBS coordination is especially relevant within VHA, where much of HCBS delivery occurs through contracted community agencies, making coordinated communication and referral pathways essential to ensure Veterans can obtain needed services [28]. Telehealth modalities used within the context of standard primary care, specialty care, or home‐based primary care can be beneficial for chronic disease monitoring, acute symptom triage, care planning/coordination, and overcoming rural access barriers [48, 49, 50]. Even with effective coordination, however, much of the essential assistance provided through HCBS—including help with bathing, cooking, household chores, and caregiver respite—can only be provided in‐person [51, 52]. Yet, the HCBS workforce has not kept pace with growing demand, with reports of accelerated turnover among direct support professionals during and after the COVID‐19 pandemic [51, 52]. Kreider and colleagues examined workforce supply relative to demand among Medicaid‐sponsored HCBS programs and found the number of home care workers per 100 HCBS participants declined by 11.6% between 2013 and 2019 [52]. Therefore, new approaches to support older adults to remain in their homes and communities as they age may be needed.

Some have suggested expanded roles for community‐based resource utilization, including community health workers, faith‐based organizations, and county‐level resources, as adjuncts to VHA‐provided HCBS [53]. Although rural residents often experience greater socioeconomic disadvantage (as observed in this analysis), including less access to services, strategies such as these may be well‐suited to address rural HCBS access gaps, given the strong social ties in many rural communities [54]. The VHA has already implemented similar models through established collaborations with community agencies—for example, the Veteran‐directed care program (previously noted), which partners with Area Agencies on Aging to deliver consumer‐directed HCBS [39] and the Veteran Community Partnerships initiative [55], which formalizes local partnerships between VAMCs and community organizations to improve service coordination and access for Veterans [56]. These examples highlight how leveraging community partnerships can help the VHA address HCBS access gaps, particularly in rural settings.

### 4.1 Strengths and Limitations

A major strength of this study is the large integrated health system of the VHA, which provides a common data platform and a broad suite of HCBS. However, focusing on Veterans limits generalizability to other healthcare settings. Although many VHA HCBS are delivered by community agencies that also serve the general public, HCBS funding models differ substantially outside VHA, so patterns of access and use may not align across systems. Our novel assignment of Veterans to VAMCs underscores that administrative geographic boundaries may not always reflect where Veterans actually receive care.

While facility fixed effects helped account for unobserved differences across facilities—including variation in HCBS capacity—future work could incorporate explicit measures of capacity such as staffing levels, contracted provider availability, and authorization volumes, as well as organizational factors like leadership priorities and clinicians' beliefs about HCBS. The RUCA‐based definition of rurality may also miss important differences in distance or geographic isolation, suggesting that incorporating travel time or distance could improve HCBS access measures. Finally, given evidence of racial and ethnic differences in unmet HCBS needs outside VHA [10, 55], it will also be important for future work to assess potential effect modification based on race and ethnicity, as well as whether rural–urban differences in HCBS use vary in direction and magnitude based on specific service type [6, 10].

### 4.2 Conclusions

This analysis found that rural Veterans were less likely than urban Veterans to receive HCBS, but these findings varied considerably across VAMCs nationally. Importantly, at each level of facility rurality, some VAMCs found ways to overcome the challenges of delivering HCBS in rural settings, ensuring Veterans' access to HCBS regardless of their residential location. As the need for HCBS continues to grow among the Veteran population, learning from those VAMCs that successfully deliver HCBS to both rural and urban Veterans could be a mechanism to improve HCBS delivery nationally.

## Author Contributions

All significant contributors to this work are listed as authors. All authors have reviewed and approved the final submission. Specific contributions: H.D.: data interpretation, manuscript preparation. D.H.: data acquisition, analysis and interpretation, manuscript preparation. F.D.: data acquisition, analysis and interpretation, manuscript preparation. C.W.H.: data acquisition, analysis and interpretation, manuscript preparation. M.A.M.: data interpretation, manuscript preparation. S.L.S.: data interpretation, manuscript preparation. R.N.A.: data interpretation, manuscript preparation. M.L.B.: data interpretation, manuscript preparation. C.B.: data interpretation, manuscript preparation. T.P.: data interpretation, manuscript preparation. D.C.: data interpretation, manuscript preparation. J.L.S.: conception and design, data interpretation, manuscript preparation. J.L.R.: conception and design; data acquisition, analysis, and interpretation; manuscript preparation.

## Funding

This study was funded by the US Department of Veterans Affairs (VA), Health Systems Research (HSR), Quality Enhancement Research Initiative (QUERI) through the Charleston, Providence, and Iowa City Evidence‐Based Policy Evaluation Center (CPIC) (EBP 22‐105). Drs. Heather Davila, Michelle A. Mengeling, and Samantha L. Solimeo receive support from the VHA Office of Rural Health Veterans, Rural Health Resource Center‐Iowa City (#0000042).

## Disclosure

All authors are employees of the US Department of Veterans Affairs (VA)—Veterans Health Administration (VHA). The VHA had no role in the analysis or interpretation of data or the decision to report these data in a peer‐reviewed journal. The views expressed in this manuscript are those of the authors and do not necessarily reflect the position or policy of the VHA or the United States government.

## Conflicts of Interest

The authors declare no conflicts of interest.

## 5 Linked Article

This publication is linked to a related editorial by Romil R. Parikh. To view this article, visit https://doi.org/10.1111/jgs.70584.

## Supporting information


**Data S1:** jgs70500‐sup‐0001‐Supinfo.pdf.
