# Supplementary material for: Rural–Urban Variability in Home and Community‐Based Service Use Among Veterans
Source: J Am Geriatr Soc. 2026 Jun 10;74(9):2546–55. doi: 10.1111/jgs.70500 (PMC13589664; doi:10.1111/jgs.70500)
Supplement: Supplementary file 1 [file JGS-74-2546-s001.pdf]

Supplemental Materials – Description of Home and Community-Based Services Included in the Analysis

Service	Description	Data notes
Homemaker and Home Health Aide	Trained aides provide assistance with daily living activities and personal care services to help Veterans remain independent in their home. Assistance can include help bathing, dressing, meal preparation, eating, and light housework. Most homemaker home health aides work for community agencies contracted with VA but are supervised by a registered nurse.	Primary VHA stop codes: 190, 191, 658 OR Secondary stop codes: 190<br><br>Integrated Veteran Care-Consolidated Data Set (IVD-CDS) category of care: 'HOMEMAKER/HOME HEALTH AIDE', 'NIC HOMEMAKER/HOME HEALTH AIDE',
VA-Paid Respite Care	Offers short-term care at home or at an adult day care program to give family caregivers a break. VA-Paid respite care is delivered through contract agencies in in the community.	Primary VHA stop codes: 190, 191, 658 OR Secondary stop codes: 190<br><br>IVD-CDS category of care: 'NIC RESPITE CARE',
VA-Paid Adult Day Healthcare	Allows Veterans who are socially isolated or need help with activities of daily living to engage in social activities, peer support, companionship, and recreation during the day. Adult day healthcare may provide respite for caregivers. VA-paid adult day healthcare is delivered through contract community agencies.	Primary VHA stop codes: 190, 191, 658 OR Secondary stop codes: 190<br><br>IVC-CDS category of care: 'ADULT DAY HEALTH CARE', 'NIC ADULT DAY CARE'
Veteran-Directed Care	Provides a flexible budget for Veterans with functional or cognitive limitations to manage their own care services independently or with help from a family care partner. Services, equipment, and monthly stipends are provided to Veterans with support from counselors and VA staff.	Primary VHA stop codes: 190, 191, 658 OR Secondary stop codes: 190<br><br>Plus IVC-CDS category of care: 'VETERAN DIRECTED CARE'
VA-Paid Skilled Home Care	Provides nursing care and other health services in Veterans' homes through a VA-contracted community-based agency to those living far from a VA medical facility. In addition to nursing services, purchased skilled home healthcare can include	Primary stop codes: 680, 681 OR Secondary stop codes: 680<br><br>Plus IVC-CDS category of care: NIC SKILLED HOME CARE', 'NIC SKILLED

	physical therapy, occupational therapy, speech therapy, or social work services.	NURSING', 'SKILLED HOME HEALTH CARE', 'NIC IV THERAPY/INFUSION',
Home Based Primary Care	Delivers long-term primary care and healthcare services to Veterans with complex health conditions and needs and with high risk of hospitalization and nursing home care. Home based primary care is delivered directly within the VA and utilizes an interdisciplinary team and unified care plan that is supervised by a VA physician.	Primary Stop codes: 156, 157, 170, 171, 172, 173, 174, 176, 177<br><br>HBPC telehealth:<br>Primary Stop codes: 178<br><br>HBPC video:<br>Primary Stop codes: 156, 157, 170, 171, 172, 173, 174, 176, 177, 178 AND<br>Secondary stop codes: 179 (CVT) or 690 (VVC)

The Integrated Veteran Care-Consolidated Data Set is used to identify services paid for by the Veterans Health Administration but delivered through contract community agencies.
